# Molecular subtypes based on DNA methylation predict prognosis in colon adenocarcinoma patients

**DOI:** 10.18632/aging.102492

**Published:** 2019-12-18

**Authors:** Changshun Yang, Yu Zhang, Xiaoqin Xu, Weihua Li

**Affiliations:** 1 Department of Surgical Oncology, Fujian Provincial Hospital, Fuzhou 350001, China; 2 Department of Pathology, The First Affiliated Hospital of Fujian Medical University, Fuzhou 350001, China; 3 School of Public Health, Fujian Medical University, Fuzhou 350001, China

**Keywords:** DNA methylation, colon adenocarcinoma, TCGA database, prognostic prediction model, bioinformatics

## Abstract

Tumor heterogeneity makes early diagnosis and effective treatment of colon adenocarcinoma difficult. As an important regulator of gene expression, DNA methylation can influence tumor heterogeneity. In this study, we explored the prognostic value of subtypes based on DNA methylation status in 424 colon adenocarcinoma samples from the Cancer Genome Atlas database. Differences in DNA methylation levels were associated with differences in T, N, and M category, age, stage, and prognosis. Seven subgroups were identified based on consensus clustering using 356 CpG sites that significantly influenced survival. Finally, a prognostic model was constructed and used to classify samples in a testing dataset into seven DNA methylation subgroups based on the classification results of a training dataset. These specific classifications based on DNA methylation may help account for heterogeneity within previously established molecular subgroups of colon adenocarcinoma and could potentially aid in the development of more effective personalized treatments.

## INTRODUCTION

Colon cancer, which is the fourth most common cancer worldwide and the fifth leading cause of cancer-related death [1], is especially prevalent among middle-aged and elderly people [2]. In recent years, colon cancer morbidity and mortality rates have increased due to changes in diet and environment. An estimated two-thirds of colon adenocarcinoma patients have advanced stage disease upon diagnosis [3]. Colon cancer is highly heterogeneous, and differences in sensitivity to chemotherapy among clinical subtypes result in a variety of prognostic outcomes.

Colon adenocarcinoma usually originates from epithelial dysplasia in the colonic mucosa followed by malignant infiltration and growth [4]. Studies have demonstrated that epigenetic changes are closely associated with the onset, development, and malignant transformation of colon adenocarcinoma [5]. Epigenetic mechanisms that regulate gene activity have received much attention in post-genomic era research [6, 7]. One such mechanism, DNA methylation, occurs early and frequently during the complex process of oncogenesis. DNA methylation changes accumulate as the disease progresses and are now considered telltale signs of malignant colon adenocarcinoma [8]. Identification of specific epigenetic biomarkers in samples from colon adenocarcinoma patients might aid in the development of personalized treatment plans. Such biomarkers could play a key role in prognostic evaluation, staging, relapse prediction, and timely initiation of appropriate therapeutic drugs and interventions.

DNA methylation refers to the transfer of the methyl group of S-adenosylmethionine (SAM) to the number five carbon atom of cytosine to form 5-methylcytosine (5-mC). This process is catalyzed by DNA methyltransferase (DNMT) and mostly occurs in CpG structures. Unmethylated CpGs will cluster to form CpG islands at the core sequence and transcription start site (TSS) in the structural gene promoter [9]. Chromosomal instability (CIN) and microsatellite instability (MSI) are both involved in the development of colon adenocarcinoma [10, 11]. Ubiquitous hypomethylation can activate proto-oncogenes and lead to CIN, and hypermethylation of CpG islands in specific regions may inhibit the expression of tumor suppressor genes (TSG), DNA repair genes, housekeeping genes, and cell cycle control genes [12]. Currently, methylation of several promoter sequences, including MGMT, MLH1, APC1A, SHOX2, RASSF1A, and PHD1, has been associated with the onset and development of colon adenocarcinoma [13–16]. Nevertheless, specific methylation sequences in the promoter regions of these genes have not yet been identified. In addition, the clinical significance of methylation of these genes in relation to tumor classification, survival time, and prognosis has not yet been examined in large groups of colon adenocarcinoma patients. In this study, we therefore developed a prognostic prediction model that integrates multiple DNA methylation biomarkers based on high-throughput omics data to improve clinical prognostic evaluation and personalized treatments.

## RESULTS

### Identification of potential prognostic methylation sites associated with OS in training dataset patients

After patient data were preprocessed as described in Materials and Methods, 22,830 methylation sites were identified. We then divided the patients into training (Supplementary Table 2, clinical information in Supplementary Table 3) and testing datasets (Supplementary Table 4, clinical information in Supplementary Table 5). Of the 22,830 methylation sites, 864 CpG sites were identified as potential DNA methylation biomarkers for overall survival in colon adenocarcinoma (COAD) patients using univariate Cox regression analysis (Supplementary Table 6, *P*<0.05). Univariate Cox proportional-hazards regression analysis revealed that T category (primary tumor), N category (regional lymph nodes), M category (distant metastasis), stage, and age were significantly associated with overall survival (respective log-rank *P* values: 6.499e-07, 1.572e-06, 1.769e-07, 8.524e-07, and 0.04611). A subsequent multivariate Cox regression analysis of the 864 methylation sites with T category, N category, M category, stage, and age as covariates identified 356 independent prognosis-associated CpG sites. These 356 sites were considered potential prognostic methylation sites (Supplementary Table 7).

### Consensus clustering to identify distinct DNA methylation prognosis subgroups and intercluster prognosis analysis

**Figure 1:**
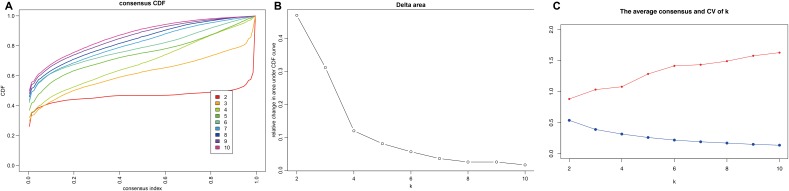
**Criteria for selecting number of categories.** (**A**) Consensus among clusters for each category number k. (**B**) Delta area curves for consensus clustering indicating the relative change in area under the cumulative distribution function (CDF) curve for each category number k compared to k-1. The horizontal axis represents the category number k and the vertical axis represents the relative change in area under CDF curve. (**C**) The average cluster consensus and coefficient of variation among clusters for each category number k. The blue line represents the average cluster consensus and the red line represents the coefficient of variation among clusters.

**Figure 2:**
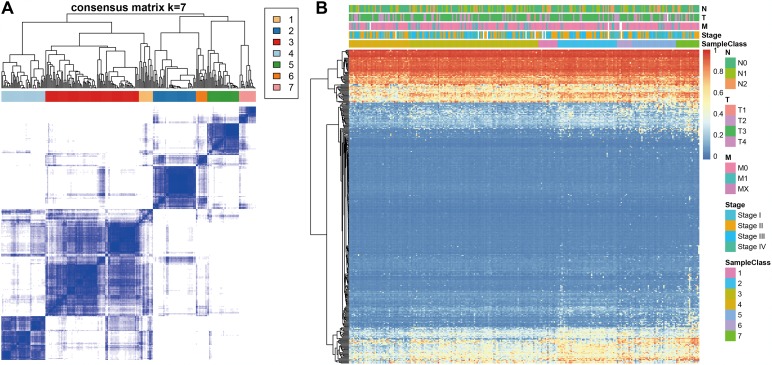
**Consensus matrix for DNA methylation classification with the corresponding heat map.** (**A**) Color-coded heatmap corresponding to the consensus matrix for k=7 obtained by applying consensus clustering. Color gradients represent consensus values from 0–1; white corresponds to 0 and dark blue to 1. (**B**) A heatmap corresponding to the dendrogram in (**A**) was generated using the heatmap function with DNA methylation classification, TNM stage, clinicopathological stage, and histological type as the annotations.

Consensus clustering of the 356 potential prognostic methylation sites was used to identify distinct DNA methylation molecular subgroups of COAD for prognostic purposes. Numbers of clusters were determined according to the following criteria: relatively high consistency within the cluster, relatively low coefficient of variation (Figure 1C), and no appreciable increase in the area under the CDF curve. We calculated average cluster consensus and the coefficient of variation among clusters depending on category number. The area under the Cumulative Distribution Function (CDF) curve began to stabilize after 5 categories (Figure 1A and 1B). To improve the prognostic value of the COAD classifications, we choose larger cluster numbers when possible. A consensus matrix was also a used as described in Materials and Methods to help determine the optimal number of clusters. The consensus matrix shown in Figure 2A represents the consensus for k=7 and displays a well-defined 7-block structure. A heatmap corresponding to the dendrogram in Figure 2A with T category, N category, M category, stage, age, and DNA methylation subgroup as the annotations is shown in Figure 2B.

**Figure 3:**
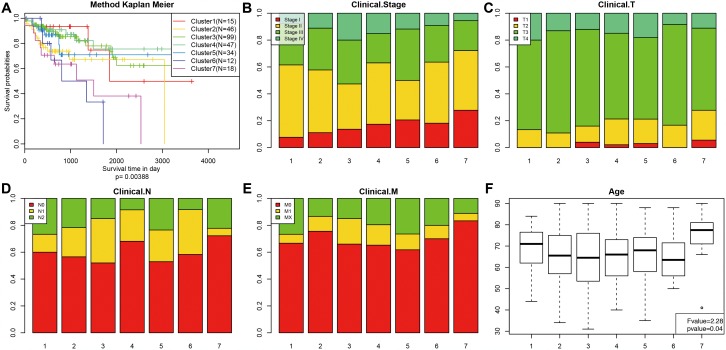
**Comparison of prognosis, TNM stage, grade, and age between the DNA methylation clusters.** (**A**) Survival curves for each DNA methylation subtype in the training set. The horizontal axis represents survival time (days), and the vertical axis represents the probability of survival. The number of samples in each cluster is shown in parentheses in the legend. The log-rank test was used to assess the statistical significance of differences between subtypes. Stage score (**B**), topography score (**C**), lymphocyte infiltration (**D**), metastasis (**E**), and age (**F**) distributions for each DNA methylation subtype in the training set. The horizontal axis represents the DNA methylation clusters.

Kaplan-Meier survival analysis revealed significant differences in prognosis among the 7 clusters (P<0.05). As shown in Figure 3A, Clusters 3 and 4 had the best prognoses, while Cluster 7 had the worst. We then analyzed intracluster proportions for the 7 clusters according to T category, N category, M category, stage, and age as shown in Figure 3B–3F, respectively. Tendencies for associations between characteristics and specific clusters were as follows: Clusters 3 and 5 with advanced stage; Clusters 4, 5, and 7 with lower T grade; Clusters 4 and 7 with lower N grade; Clusters 3, 4, and 5 with higher M grade; Cluster 7 with older age (Figure 3F). These results indicate that each clinical parameter was associated with a different intra-cluster proportion.

### Identifying different characteristics based on DNA methylation clustering and screening of cluster-specific methylation sites

**Figure 4:**
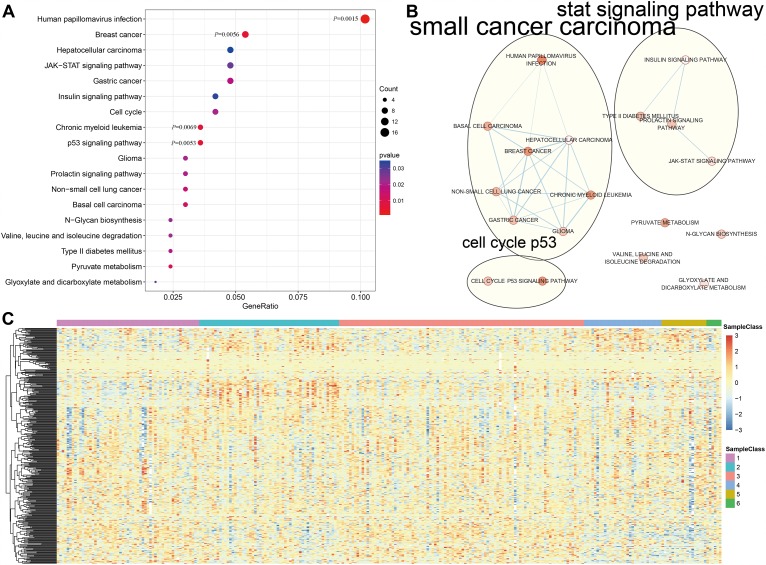
**Gene annotations of 356 methylated sites.** (**A**) KEGG function enrichment analysis of annotated genes for the 356 CpG sites. (**B**) Crosstalk analysis of the enriched KEGG pathways using Enrichment Map Cytoscape plugin. (**C**) Cluster analysis heat map for annotated genes associated with the 376 CpG sites.

Genome annotations for the 356 CpG sites described above were used to identify a total of 415 corresponding promotor genes. We then conducted functional enrichment analysis of these 415 genes and identified 18 significantly enriched pathways (*P*<0.05) as shown in Figure 4A and Supplementary Table 8. The three most significantly enriched pathways were human papillomavirus infection, p53 signaling, and a breast cancer pathway. Crosstalk analysis was then performed on the 18 pathways using the Enrichment Map Cytoscape plugin [17]; Jaccard Indexes and Overlap Coefficients were calculated to analyze pairwise relationships among pathways. As shown in Figure 4B, close relationships were identified among the 18 pathways and between those pathways and cell cycle and p53 signaling pathways when the Jaccard Index was >0.375.

We then explored the expression of the methylated genes identified in the subgroups. Expression values were available for 376 of the 415 genes in the 266 training expression dataset samples. The gene expression heatmap is shown in Figure 4C, and the raw data is shown in Supplementary Table 9. Gene expression patterns differed among the subgroups, suggesting that DNA methylation levels were generally reflective of expression for these genes.

**Figure 5:**
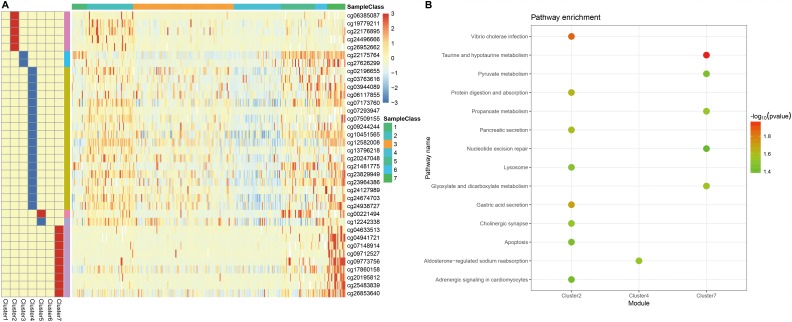
**Specific hyper/hypo-methylation CpG sites for each DNA methylation cluster.** (**A**) Specific CpG sites are shown for each DNA methylation prognosis subtype. Red and blue bars represent hyper- and hypomethylation CpG sites, respectively. (**B**) KEGG pathway enrichment analysis of specific CpG sites.

**Figure 6:**
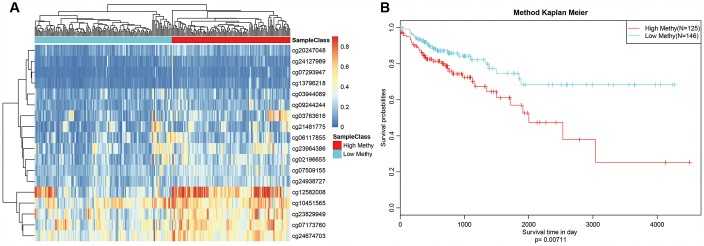
**Box plot of CpG methylation levels of the 7 Clusters.** Cluster 4 has the lowest CpG methylation level.

Next, we screened for cluster-specific methylation sites by including the methylation sites as characteristics of the clusters. First, differences among the 7 clusters were analyzed for every methylation site as described in Materials and Methods; the results are shown in Supplementary Table 10. Ultimately, 36 cluster-specific methylation sites shown in Supplementary Table 11 and the heatmap in Figure 5A were identified. Cluster 4 had the largest number of specific sites, all of which were hypomethylated, and the methylation level was the lowest among all the clusters (Figure 6). Genome annotations of the 36 specific sites were used to identify their corresponding genes (Supplementary Table 12). Analysis using clusterProfiler indicated that these genes were enriched in 14 pathways as shown in Figure 5B (Supplementary Table 13). These 14 pathways were only enriched in Clusters 2, 4, and 7; apoptosis, secretion, and other pathways were enriched in Cluster 2, the aldosterone-regulated sodium reabsorption pathway was enriched in Cluster 4, and multiple metabolic pathways were enriched in Cluster 7. These results indicated each cluster had unique gene expression and pathway characteristics.

### Constructing and evaluating the COAD prognosis prediction model

**Figure 7:**
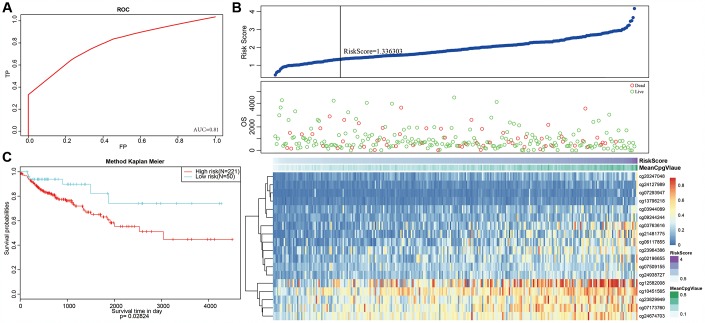
**Methylation sites may predict prognosis in colon adenocarcinoma.** (**A**) Reclustered samples with hierarchical analysis separated into hypomethylation and hypermethylation groups. (**B**) Analysis of prognostic differences between hypomethylation and hypermethylation groups.

We selected Cluster 4 as the seed cluster because it included a large number of samples, was associated with good prognosis, and had the largest number of specific methylation sites. Cluster 4 had 18 specific methylation sites, all of which were hypomethylated. Methylation level profiles for these 18 specific sites were obtained for all samples, which were then re-clustered using hierarchical cluster analysis. The samples were divided into hypermethylation and hypomethylation groups as shown in Figure 7A. Prognosis analysis revealed significant differences between the two groups (Figure 7B). Specifically, prognoses were worse in the hypermethylation group, indicating that these specific methylation sites might serve as prognostic markers.

**Figure 8:**
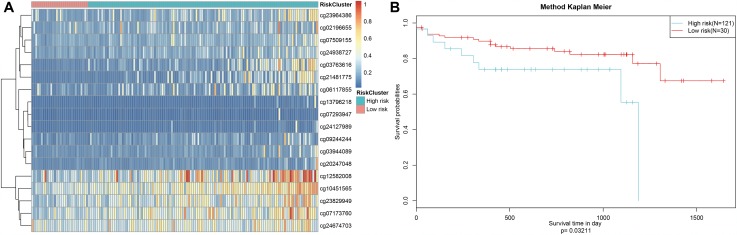
**Construction of the prognosis prediction model for training set colon adenocarcinoma patients.** (**A**) ROC curves of prognostic predictors in colon adenocarcinoma patients. (**B**) The horizontal axis represents the samples, and the vertical axis represents risk scores (top), overall survival (middle), and methylation site (bottom). (**C**) Analysis of prognostic differences after classification in the training set.

Next, we constructed a Cox Proportional Hazard Model based on methylation level profiles for the 18 specific sites combined with prognosis information using the formula provided in Materials and Methods. The results of ROC analyses performed using risk scores calculated for each sample are shown in Figure 8A. The area under curve (AUC) was 0.81, indicating that the model functioned well. The samples were then ordered by risk score to determine whether methylation level varied systematically with risk score (Figure 8B). Methylation levels for the 18 specific sites significantly increased as risk scores increased. Moreover, the 50 samples with the lowest risk scores also had significantly lower. methylation levels than the other samples, which was consistent with the cluster analysis.

A risk score cut-off value of 1.336303 (Figure 8B) was used to divide the samples into high and low risk groups. Hypomethylation was associated with low-risk patients, while hypermethylation was associated with high-risk patients. Furthermore, prognoses differed significantly between the two groups as shown in Figure 8C.

**Figure 9:**
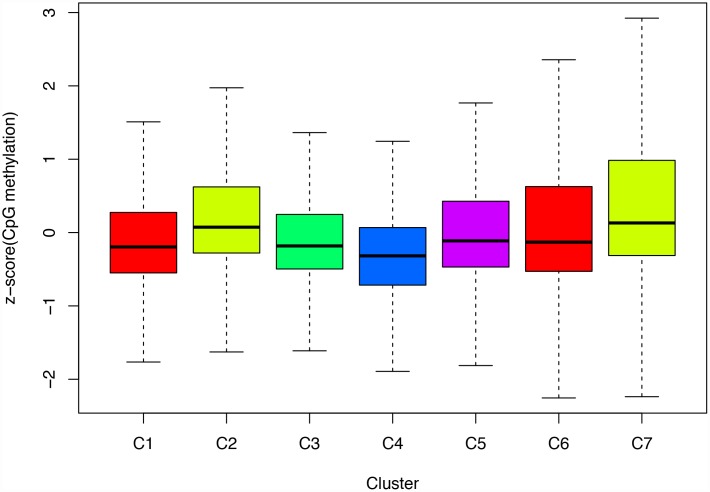
**Stability of the prognosis prediction model in testing set colon adenocarcinoma patients.** (**A**) The horizontal axis represents the samples, and the vertical axis represents methylation site. (**B**) Analysis of prognostic differences after classification in the testing set.

Finally, the prognostic model was used to predict outcomes in testing dataset patients. Methylation level profiles for the 18 CpG sites were obtained for testing dataset samples and risk scores were calculated using the prognostic model. Sorting the samples by risk score produced the heatmap shown in Figure 9A, which indicated that risk scores increased as methylation levels increased. Testing dataset samples were then divided into high-risk and low-risk groups using the cut-off score of 1.336303. Prognoses again differed significantly between the two groups (Figure 9B, *P*=0.0321). These results were consistent with those obtained from the training dataset, demonstrating the predictive accuracy and stability of our model.

## DISCUSSION

Colon adenocarcinoma is one of the most common gastrointestinal tract carcinomas and its incidence is increasing year over year, at least in part due to changes in dietary behaviors [18]. In most cases, colon adenocarcinoma is diagnosed in advanced stages and is therefore associated with unfavorable prognoses and poor 5-year survival rates, especially in patients with distant metastases [19, 20]. Although 5-year survival rates for colon adenocarcinoma have improved in recent years due to advancements in surgical treatments, radiotherapies, and chemotherapies, they remain unsatisfactory. In order to improve the management of colon adenocarcinoma, it is important to identify novel clinical biomarkers that can improve prognostic evaluation, molecular subtyping, staging, recurrence prediction, and the success of early interventions and medication. More recently, epigenetic changes, including universal hypomethylation, hyper- methylation of key TSGs, and histone modifications have been observed in all stages of colon adenocarcinoma. Abnormally methylated genes can likely serve as non-invasive biomarkers for early detection, diagnosis, treatment selection, response evaluation, and potential use of new therapies. Several studies have reported that ubiquitous hypo- and hypermethylation of CpG islands (CGIs) in specific promoter regions play key roles in the onset and development of colon adenocarcinoma. Ubiquitous hypomethylation can activate proto-oncogenes at the initial stages of oncogenesis and induce CIN and MSI. These events are also associated with external factors such as environment and nutrition [18, 21]. Hypermethylation of CGIs also play an important role in the development of colon adenocarcinoma. In addition, hypermethylation of tumor-inhibiting factor, adhesion molecule, and angiogenesis inhibitor genes, as well as other important bioactivators, reduces or silences their expression, thereby promoting tumor progression and distant metastases.

Several promoter sequence-specific methylations associated with the onset and development of colon adenocarcinoma have recently been discovered. Some studies have demonstrated that epigenetic changes occur long before genetic changes in colon adenocarcinoma. Abnormal DNA methylation occurs very early in oncogenic processes during the development of precancerous lesions in histologically normal colonic mucosal tissues [22–24]. Therefore, identification of epigenetic changes alone or in combination with detection of other standard biomarkers can be performed during early diagnosis of colon adenocarcinoma. Methylation of the ESR1, MGMT, HPP1/TPEF, HLTF, and NGFR genes [25–28] is associated with the onset of colon adenocarcinoma and occurs during the early stages of oncogenesis. Furthermore, markers indicative of hypermethylation of certain genes can be detected in blood, urine, and stool samples. Recent studies have also demonstrated that methylation patterns can be used for disease staging and prognostic evaluation. For example, methylation of APC1A, CHD1, DKK3, and MYOD is associated with prognosis in colon adenocarcinoma patients [15].

Although methylation may serve as an important biomarker in colon adenocarcinoma, the specific methylation sequences in the promoter regions of the affected genes remain unknown. Additionally, the clinical and statistical significance of methylation of these genes in relation to tumor classification, survival time, and prognosis need to be confirmed in larger groups of COAD patients. We attempted to address these issues in this study by developing a classification method that integrated several DNA methylation biomarkers for prognostic evaluation of therapeutic efficacy and to help guide treatment selection. The model can facilitate identification of new biomarkers, targets for precision medicine, and disease molecular subtype classification in COAD patients. The model may also help in prognostic prediction, clinical diagnosis, and management of patients with different epigenetic subtypes of COAD.

However, there are some limitations in the study. First, the prognosis prediction model needs further validation in our own independent tissue samples. Second, in practice, construction of a prognostic prediction model is far more complicated and need platform or other tools. While our study aimed to investigate the possibility to construct a prognostic prediction model, so it was rudiment, and needs improved. Third, it’s a very difficult work in judging optimal k in consensus. In conclusion, based on the TCGA database and a series of bioinformatics approaches, we have identified prognosis-specific methylation sites and constructed a prognostic prediction model for colon adenocarcinoma patients. This model can facilitate identification of new biomarkers, targets for precision medicine, and disease molecular subtype classification in colon adenocarcinoma patients. Thus, the model may aid in prognostic prediction, clinical diagnosis, and management of patients with different epigenetic subtypes of colon adenocarcinoma.

## MATERIALS AND METHODS

### Data selection and pre-processing

RNA-sequencing data from 519 primary colon adenocarcinoma samples were downloaded from the TCGA data portal (https://cancergenome.nih.gov/, 2018-08-13). Clinical information for these samples, including follow-up data for 459 patients, is shown in Supplementary Table 1. Methylation data from Illumina Infinium HumanMethylation450 and 27 BeadChip arrays performed on samples from 337 and 203 patients, respectively, were downloaded from the UCSC Cancer Browser.

Only data from samples with clinical follow-up times of more than 30 days were included in this study. The methylation level of each site was represented by the β-value, which ranges from 0 (unmethylated) to 1 (fully methylated). CpG sites for which data was missing in more than 70% of the samples were excluded from analysis. Cross-reactive genome CpG sites as defined in “Discovery of cross-reactive probes and polymorphic CpG in the Illumina Infinium HumanMethylation450 microarray” were also excluded. Remaining sites with for which data were not available (NAs) were imputed using the k-nearest neighbors (KNN) imputation procedure. The ComBat algorithm in the sva R package [29] was used to remove batch effects by integrating all DNA methylation array data and incorporating batch and patient information. Unstable genomic sites, including CpGs in sex chromosomes and single nucleotide polymorphisms, were removed. Because DNA methylation in promoter regions strongly influences gene expression, we specifically examined CpGs in promotor regions. Promoter regions were defined as 2 kb upstream to 0.5 kb downstream of transcription start sites. Finally, we selected samples for which gene expression profiles were available. In total, 424 samples and 22,830 methylation sites were included in subsequent analyses.

The samples were divided into 2 cohorts: a training set (data from HumanMethylation 450 BeadChip) and a testing set (data from HumanMethylation 27 BeadChip). Methylation site profiles and clinical information for the training set (age, TNM staging, grade, gender, and survival time) are shown in Supplementary Tables 2 and 3, respectively. Testing set methylation site profiles and clinical information are shown in Supplementary Tables 4 and 5, respectively.

### Determining classification features using COX proportional risk regression models

Because COAD molecular subtype seemed to influence prognosis in the samples used in this study, CpG sites that significantly influenced survival were used as classification features. First, univariate COX proportional risk regression models were constructed using methylation level for each CpG site, T category, N category, M category, age, stage, gender, and survival data. The significant CpGs obtained from univariate COX proportional risk regression models were then introduced into multivariate COX proportional risk regression models using T category, N category, M category, age, and stage, which were also significant in the univariate models, as covariates. Finally, CpG sites that were significant in both univariate and multivariate Cox regression analyses were selected as characteristic CpG sites (Supplementary Table 7).

### Identification of molecular subtypes associated with prognosis using consensus clustering

Consensus clustering was performed using the ConsensusClusterPlus package in R [30] to identify COAD subgroups based on the most variable CpG sites. The algorithm began by subsampling a proportion of items and features from the data matrix where each subsample was partitioned into up to k groups by k-means. This process was repeated for a user-specified number of repetitions; these multiple clustering algorithm runs were used to establish consensus values and to assess the stability of the identified clusters. Pairwise consensus values, defined as the proportion of clustering runs in which two items are grouped together, were calculated and stored in a consensus matrix for each k. Then, for each k, a final agglomerative hierarchical consensus clustering using a distance of 1-consensus values was completed and pruned to k groups. This algorithm determined “consensus” clustering by measuring the stability of clustering results from the application of a given clustering method to random subsets of data. In each iteration, 80% of the tumors were sampled, and the k-means algorithm with the Euclidean squared distance metric was used. These results were compiled over 100 iterations. After executing ConsensusClusterPlus, we obtained the cluster consensus and item-consensus results. Graphical output results included heatmaps of the consensus matrices, which displayed the clustering results, consensus cumulative distribution function (CDF) plots, and delta area plots, and which allowed us to determine an approximate number of clusters. Numbers of clusters were determined according to the following criteria: relatively high consistency within the cluster, relatively low coefficient of variation, and no appreciable increase in the area under the CDF curve. The coefficient of variation was calculated according to the following formula: CV = (SD/MN)*100%, in which SD represents the standard deviation and MN represents the average number of samples. The category number was defined as the area under the CDF curve and showed no significant change. In order to generate more detailed classification categories for COAD, larger numbers of categories were favored.

The heatmap corresponding to the consensus clustering was generated by the pheatmap R package. Consensus values from 0 (white) to 1 (dark blue) are depicted using a color gradient; the matrix is arranged so that items belonging to the same cluster are adjacent to each other. In this arrangement, a matrix corresponding to a perfect consensus will show a color-coded heatmap characterized by blue blocks along the diagonal on a white background. The color-coded heatmap corresponding to the consensus matrix obtained by applying consensus clustering to these cases is shown in Figure 2A and represents the consensus for k = 7, which displays a well-defined 7-block structure.

### Survival and clinical characteristics analyses

Kaplan–Meier plots were used to illustrate overall survival among COAD subgroups defined by DNA methylation profiles. The log-rank test was used to evaluate the significance of differences among the clusters. Survival analyses were performed using the survival package in R. Associations between both clinical and biological characteristics and DNA methylation clustering were analyzed using the chi-squared test. All tests were two-sided; *P*<0.05 was considered statistically significant for all tests.

### Functional enrichment analysis and genome annotation

We used the clusterProfiler package in R [31] combined with KEGG to perform gene enrichment analysis of the Gene Ontology, Biological Pathways, and Regulatory motifs in DNA and Protein gene groups.

### Construction and testing of the prognostic prediction model

The coxph function of the survival package in R was used to construct a Cox Proportional Hazard Model based on the combination of methylation profiles for 18 CpG sites and prognostic information. The formula used for this model was: Risk Score=0.12*cg02196655 +1.35*cg03763616+0.73*cg03944089+0.73*cg0611 78 55+0.76*cg07173760-3.96*cg07293947-0.76*cg07509 155+0.58*cg09244244+0.4*cg10451565+0.28*cg12582008+1.99*cg13796218+3.6*cg20247048+1.34*cg21481775+0.42*cg23829949-0.28*cg23964386+0.96*cg 24127989-0.45*cg24674703+0.84*cg24938727.

## Supplementary Material

Supplementary Table 1

Supplementary Table 2

Supplementary Table 3

Supplementary Table 4

Supplementary Table 5

Supplementary Table 6

Supplementary Table 7

Supplementary Table 8

Supplementary Table 9

Supplementary Table 10

Supplementary Table 11

Supplementary Tables 12 and 13
