# Supplementary material for: Molecular subtypes based on DNA methylation predict prognosis in colon adenocarcinoma patients
Source: Aging (Albany NY). 2019 Dec 18;11(24):11880–92. doi: 10.18632/aging.102492 (PMC6949097; doi:10.18632/aging.102492)
Supplement: Supplementary Tables 12 and 13 [file aging-11-102492-s002..pdf]

Supplementary Table 12. Genome annotations of the 36 cluster-specific methylation sites.

CpG	Chrom	Start	End	GeneSymbol	Feature_Type
cg07148914	chr20	34873032	34873033	ACSS2	Island
cg06385087	chr20	59006848	59006849	CTSZ	Island
cg09712527	chr13	41194353	41194354	KBTBD7	Island
cg26853640	chr19	38389033	38389034	SPRED3	S_Shore
cg23964386	chr9	111599969	111599970	PTGR1	.
cg27626299	chr7	27242812	27242813	EVX1	Island
cg22175764	chr20	58228908	58228909	ANKRD60	Island
cg24938727	chr3	42701501	42701502	HHATL	.
cg07173760	chr19	39738626	39738627	CLC	.
cg04941721	chr4	74365184	74365185	EREG	.
cg02196655	chr2	10690638	10690639	RN7SL832P	S_Shore
cg24127989	chr7	128409454	128409455	IMPDH1	Island
cg10451565	chr19	47336874	47336875	C5AR2	.
cg09244244	chr5	95556203	95556204	TTC37	S_Shore
cg02196655	chr2	10690638	10690639	NOL10	S_Shore
cg26853640	chr19	38389033	38389034	GGN	S_Shore
cg03944089	chr6	134176241	134176242	SGK1	N_Shore
cg27626299	chr7	27242812	27242813	EVX1-AS	Island
cg12242338	chr3	140678247	140678248	TRIM42	.
cg02196655	chr2	10690638	10690639	Metazoa_SRP	S_Shore
cg07293947	chr5	175478241	175478242	SFXN1	N_Shore
cg24938727	chr3	42701501	42701502	HHATL-AS1	.
cg12582008	chr13	53029151	53029152	OLFM4	.
cg21481775	chr8	23682114	23682115	NKX3-1	N_Shore
cg26952662	chr8	103371271	103371272	CTHRC1	Island
cg24674703	chr11	61102488	61102489	CD5	.
cg25483839	chr9	107282606	107282607	RAD23B	N_Shore
cg07148914	chr20	34873032	34873033	GGT7	Island
cg23829949	chr1	244051377	244051378	ZBTB18	S_Shore
cg06117855	chr3	45026296	45026297	CLEC3B	.
cg07509155	chr1	24187922	24187923	IFNLR1	Island
cg00221494	chr13	98142339	98142340	FARP1	Island
cg09773756	chr10	97499762	97499763	MMS19	Island
cg13796218	chr1	11690606	11690607	DRAXIN	N_Shore
cg20247048	chr17	64976240	64976241	AMZ2P1	S_Shore
cg22176895	chr1	213988696	213988697	PROX1-AS1	N_Shore
cg17860158	chr1	205043441	205043442	CNTN2	.
cg22176895	chr1	213988696	213988697	PROX1	N_Shore
cg19779211	chr11	2444261	2444262	KCNQ1	Island
cg03763616	chr19	50418740	50418741	SPIB	S_Shelf
cg04633513	chr1	206117303	206117304	RP11-38J22.3	Island
cg04633513	chr1	206117303	206117304	AVPR1B	Island
cg20195812	chr2	191150951	191150952	STAT4	.
cg24496666	chr1	78046295	78046296	GIPC2	Island

Supplementary Table 13. Functional enrichment analysis and the enriched 14 pathways.

ID	Description	GeneRatio	BgRatio	pvalue	p.adjust	qvalue	gene ID	Count	CLuster
hsa05110	Vibrio cholerae infection	1/2/2019	50/7470	0.01334297	0.03818514	NA	3784	1	Cluster2
hsa04971	Gastric acid secretion	1/2/2019	75/7470	0.01998085	0.03818514	NA	3784	1	Cluster2
hsa04974	Protein digestion and absorption	1/2/2019	90/7470	0.02395282	0.03818514	NA	3784	1	Cluster2
hsa04972	Pancreatic secretion	1/2/2019	96/7470	0.02553935	0.03818514	NA	3784	1	Cluster2
hsa04725	Cholinergic synapse	1/2/2019	112/7470	0.02976379	0.03818514	NA	3784	1	Cluster2
hsa04142	Lysosome	1/2/2019	123/7470	0.03266277	0.03818514	NA	1522	1	Cluster2
hsa04210	Apoptosis	1/2/2019	136/7470	0.03608324	0.03818514	NA	1522	1	Cluster2
hsa04261	Adrenergic signaling in cardiomyocytes	1/2/2019	144/7470	0.03818514	0.03818514	NA	3784	1	Cluster2
hsa04960	Aldosterone-regulated sodium reabsorption	1/6/2019	37/7470	0.029363	0.16122387	0.15428122	6446	1	Cluster4
hsa00430	Taurine and hypotaurine metabolism	1/7/2019	11/7470	0.01026658	0.1738025	0.14317826	2686	1	Cluster7
hsa00630	Glyoxylate and dicarboxylate metabolism	1/7/2019	30/7470	0.02778703	0.1738025	0.14317826	55902	1	Cluster7
hsa00640	Propanoate metabolism	1/7/2019	32/7470	0.02961573	0.1738025	0.14317826	55902	1	Cluster7
hsa00620	Pyruvate metabolism	1/7/2019	39/7470	0.03599296	0.1738025	0.14317826	55902	1	Cluster7
hsa03420	Nucleotide excision repair	1/7/2019	47/7470	0.04323721	0.1738025	0.14317826	5887	1	Cluster7
